# Fluorescence Polarization of Methylene Blue as a Quantitative Marker of Breast Cancer at the Cellular Level

**DOI:** 10.1038/s41598-018-38265-0

**Published:** 2019-01-30

**Authors:** Anna N. Yaroslavsky, Xin Feng, Alona Muzikansky, Michael R. Hamblin

**Affiliations:** 1 Advanced Biophotonics Laboratory, Department of Physics and Applied Physics, University of Massachusetts, Lowell, 1 University Ave., Lowell, MA 01854 USA; 2 Wellman Center for Photomedicine, Massachusetts General Hospital, Harvard Medical School, 40 Blossom Street, Boston, MA 02114 USA; 3 Massachusetts General Hospital Biostatistics Center, Massachusetts General Hospital, 50 Staniford Street, Boston, MA 02114 USA

## Abstract

A quantitative technique to detect cancer in single cells could transform cancer diagnosis. Current cancer diagnosis utilizes histopathology, which requires tissue acquisition, extensive processing and, in most cases, relies on the qualitative morphological analysis of tissues and cells. Molecular biomarkers are only available for a few specific tumor subtypes. We discovered that the fluorescence polarization (Fpol) of Methylene Blue (MB) is significantly higher in cancer than in normal human breast tissues and cells. We confirmed that fluorescence polarization imaging did not affect the viability of the cells and yielded highly significant differences between cancer and normal cells using MB concentrations as low as 0.05 and 0.01 mg/ml. To explain this phenomenon we examined intracellular localization of MB and its fluorescence lifetime. We determined that higher fluorescence polarization of MB occurs due to its increased accumulation in mitochondria of cancer cells, as well as shorter fluorescence lifetime in cancer relative to normal cells. As quantitative MB Fpol imaging can be performed *in vivo* and in real time, it holds the potential to provide an accurate quantitative marker of cancer at the cellular level.

## Introduction

According to the American Cancer Society, more than 300,000 new cases of breast cancer were diagnosed in 2017 and more than 40,000 individuals died from this disease^1^.

The most widely used method for cancer diagnosis, hematoxylin and eosin (H&E) histopathology, relies solely on the morphology of tissue and cells. The morphological similarity between malignant and benign tissue, as well as artefacts due to extensive tissue processing, can lead to incorrect or inconclusive diagnosis^2,3^. Immunohistochemistry is a more powerful histological approach for diagnosing cancers. It utilizes specific antigen-antibody reactions to detect cancer markers. However, only a small number of cancers have known molecular markers^4^. Besides, immunohistochemistry suffers from the shortcomings common to most histological methods, such as extensive tissue processing and delayed diagnosis. Fine-needle aspiration (FNA) cytology is a faster, less invasive histological method, which yields diagnosis based on evaluation of cellular morphology. It is less likely to cause complications such as pain, bleeding, and infection^5^. However, morphological analysis of single cells is more challenging, as compared to standard histopathology due to the lack of tissue architecture. FNA evaluation exhibits low specificity and sensitivity for certain types of cells that present similar morphology^6–9^. A rapid, minimally invasive, low cost method that could provide accurate quantitative marker would be invaluable for early cancer detection. Not surprisingly, the search for highly specific and detectable signatures from cancer cells has been, and continues to be, an active area of research in pathology, microscopy, imaging, and spectroscopy^10–16^.

We developed an approach for detecting cancer at the cellular level by quantitative imaging of the fluorescence polarization (Fpol) of methylene blue (MB) in single cells. MB is an FDA-approved phenothiazinium dye that has been widely used in medicine^17–19^. Therefore, in the future, Fpol imaging could be used as *in vivo*, real-time quantitative method for detecting cancer at the cellular level. Previously, we have demonstrated that MB Fpol is higher in cancerous breast and skin tissues as compared to normal tissue^20–26^. In this work, fluorescence polarization imaging of MB was used to distinguish cultured human breast cancer cells from normal human breast epithelial cells. To validate our findings and explain this phenomenon, we also examined the subcellular localization and fluorescence lifetime of MB in the live cells.

## Results

### Fluorescence Polarization (Fpol) Imaging

**Figure 1:**
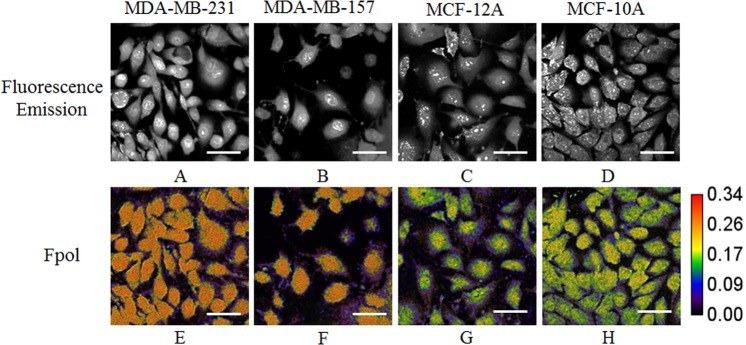
Representative MB fluorescence emission and quantitative Fpol images of MDA-MB-231, MDA-MB-157, MCF-12A, and MCF-10A cells. (**A–D**) fluorescence emission images; (**E**–**H**) Fpol images. Bar = 50 µm.

For quantitative imaging of exogenous MB fluorescence polarization, live cells were incubated with 0.05 mg/ml aqueous solution of the dye for 20 min. A multimodal confocal system simultaneously acquired co- and cross-polarized MB fluorescence images of the cells. The viability of the cells after imaging was >95%. Fpol images were processed pixel-by-pixel using the definition of fluorescence polarization. High-contrast, high-resolution fluorescence emission and fluorescence polarization imaging yielded morphological and polarization information, respectively. This enabled simultaneous analysis of MB Fpol and examination of dye localization within the cells. Representative fluorescence emission and quantitative pseudo-colored Fpol images of MDA-MB-231, MDA-MB-157, MCF-12A, and MCF-10A cells are presented in Fig. 1. Fluorescence polarization scale is shown to the right from Fig. 1H. The values of Fpol in the images range between Fpol = 0 (black color) and Fpol = 0.34 (red color). In all the cell lines, we observed accumulation of MB. In particular, nuclei, as well as some organelles outside the nucleus, exhibited high fluorescence emission. The Fpol images demonstrate notable Fpol differences between cancer (MDA-MB-231, MDA-MB-157) and normal (MCF-10A, MCF-12A) cells. Specifically, MB Fpol was higher in cancer as compared to normal cells for all cell lines investigated. Normal cells are colored green and purple (Fig. 1G,H), whereas cancer cells are mostly orange (Fig. 1E,F). It can be appreciated that Fpol is heterogeneously distributed within the normal cells, with nuclei presenting higher Fpol, as compared to the rest of the cell. The Fpol signal within cancer cells is distributed more evenly.

### Breast cancer cells exhibit higher MB Fpol than normal epithelial cells

**Figure 2:**
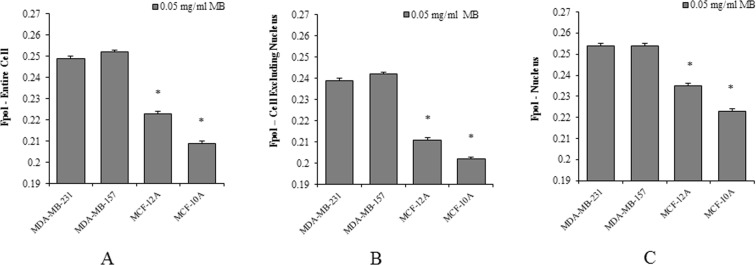
MB Fpol of MDA-MB-231, MDA-MB-157, MCF-12A, MCF-10A cells. (**A**) average MB Fpol of the entire cell; (**B**) average MB Fpol of cells with excluded nuclei; (**C**) average MB Fpol of the cell nuclei. For each cell line, Fpol value was obtained by averaging over more than 100 cells. *p < 0.0001.

To evaluate the ability of MB Fpol method to distinguish cancer and normal cells, we analyzed at least 100 cells from at least five independent experiments for each cell line. The results are summarized in Fig. 2 and summarized in Supplementary Table S1. The histograms in Fig. 2A show MB Fpol of the entire cell, averaged over all cells for each cell line investigated. In cancer cell lines, MDA-MB-231 and MDA-MB-157, average MB Fpol is 24.91 × 10^−2^ ± 0.15 × 10^−2^ and 25.16 × 10^−2^ ± 0.14 × 10^−2^, respectively. In normal cell lines, MCF-12A and MCF-10A, average MB Fpol is 22.29 × 10^−2^ ± 0.14 × 10^−2^ and 20.88 × 10^−2^ ± 0.12 × 10^−2^, respectively. The difference between MB Fpol exhibited by cancer and normal cells is highly significant (p < 0.0001). The difference between the Fpol signal from cancer versus normal cells ranged between 12% and 20%. It is the smallest between MDA-MB-231 and MCF-12A cells and the largest between MDA-MB-157 and MCF-10A cells.

Based on the observed intracellular heterogeneity of Fpol, we hypothesized that discrimination between cancer and normal cells would be facilitated by excluding cell nuclei from the analysis. To validate our hypothesis, MB Fpol analysis was repeated for the same cells with the nuclei excluded and for the cell nuclei by themselves. Indeed, larger Fpol difference between cancer and normal cells was detected when nuclei were excluded from the analysis. In particular, the average values of MB Fpol for MDA-MB-231, MDA-MB-157, MCF-12A, and MCF-10A were 23.89 × 10^−2^ ± 0.13 × 10^−2^, 24.24 × 10^−2^ ± 0.12 × 10^−2^, 21.06 × 10^−2^ ± 0.13 × 10^−2^, and 20.21 × 10^−2^ ± 0.11 × 10^−2^, respectively. The histograms of the results obtained for each cell line are shown in Fig. 2B. They demonstrate that the differences in MB Fpol values ranged between 13% and 22%. The Fpol analysis of the cell nuclei presents comparatively smaller differences. Nonetheless, cancer nuclei exhibit Fpol significantly higher than normal nuclei, but with a smaller separation ranging from 8% to 14% (Fig. 2C). Overall, a consistent and significant (p < 0.0001) MB Fpol difference between cancer and normal cells was observed. This Fpol difference is most pronounced when the cell nuclei are excluded from analysis.

### Increased accumulation of MB in mitochondria and lysosomes of cancer cells

**Figure 3:**
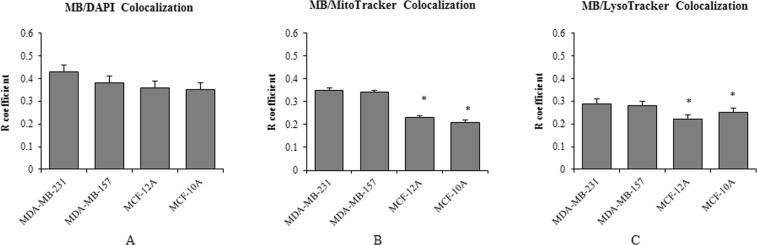
MB subcellular distribution at 0.05 mg/ml. (**A**) Degree of co-localization between MB and nuclei; (**B**) degree of co-localization between MB and mitochondria; (**C**) degree of co-localization between MB and lysosomes. Each value was obtained by averaging over at least 40 cells. *p < 0.0001.

Our imaging results indicated differences in distribution of MB Fpol between cancer and normal cells. To investigate if significantly higher MB Fpol values in cancer cells could be explained by the differences in distribution of the dye within cancerous and normal cells, we conducted co-localization experiments. According to the literature^27–31^, MB accumulates in mitochondria, lysosomes, and nuclei of the cells. We determined the degree of co-localization between MB and the above-mentioned organelles for each cell line investigated. The degree of co-localization between MB and organelle tracker (mitochondria, lysosome, nucleus) was quantified with Pearson’s Correlation Coefficient (PCC). Figure 3A–C demonstrate that MB shows the highest degree of co-localization with nucleus, followed by mitochondria and lysosomes in all the cell lines. Figure 3A demonstrates that accumulation of the dye in the nuclei is comparable among all cell lines. More importantly, our results reveal higher accumulation of MB in the mitochondria and lysosomes of cancer as compared to normal cells (Fig. 3B,C). These organelles are highly abundant in cancer cells, constituting 15% and 5% of the intracellular volume, respectively, and their matrices are more viscous than the cytosol^32,33^. Therefore, increased accumulation of MB in mitochondria and lysosomes of cancer cells offers solid explanation of the observed higher Fpol. It has been shown that MB passes through the membrane and accumulates inside the matrix of mitochondria due to its positive charge and lipophilicity^27^. Its accumulation level increases with the mitochondria membrane potential (MMP)^27,28^. In breast cancer cells, MMP is at least 60 mV higher than normal breast epithelial cells^34–38^. This correlates well with our finding and suggests that enhanced MB Fpol is associated with increased MMP in cancer cells.

### MB fluorescence lifetime is shorter in breast cancer than in normal epithelial cells

**Figure 4:**
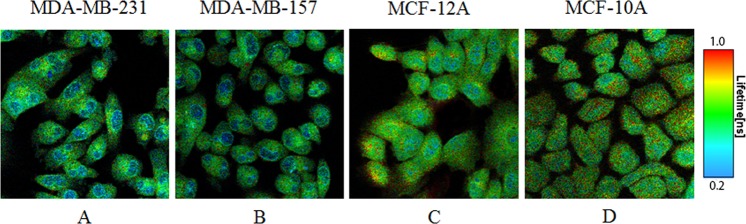
Representative fluorescence lifetime (FLIM) images of (**A**) MDA-MB-231, (**B**) MDA-MB-157, (**C**) MCF-12A, and (**D**) MCF-10A cells. Fluorescence lifetime in cancer cells is shorter in the normal cells.

**Figure 5:**
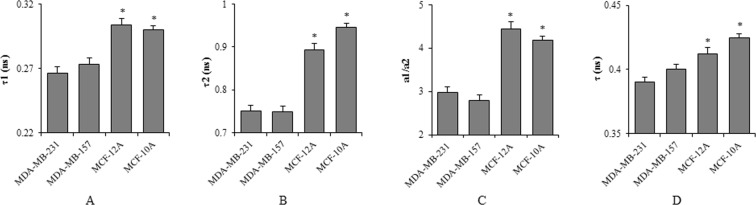
Average (**A**) τ1, (**B**) τ2, (**C**) a1/a2, and (**D**) τ values of all cell lines calculated from the biexponential decay model. For each cell line, values were averaged over at least 80 cells. *p < 0.0001.

Fpol is determined by the rotational diffusion of the fluorophore during the lifetime of the excited state^39^. Therefore, shorter fluorescence lifetimes in cancer cells could explain the higher Fpol we observed. To test this hypothesis, we examined fluorescence lifetimes exhibited by each of the investigated cell lines. Example pseudo-colored quantitative fluorescence lifetime (FLIM) images are presented in Fig. 4A–D. The scale of fluorescence lifetimes is shown to the right from Fig. 4D. The values of fluorescence lifetimes in the images range between 0.2 ns (blue color) and 1.0 ns (red color). In all the cell lines, FLIM analysis resolved two fluorescence lifetimes. The shorter lifetimes, τ1, of MDA-MB-231, MDA-MB-157, MCF-12A, and MCF-10A cells were 0.266 +/− 0.005 ns, 0.273 +/− 0.005 ns, 0.304 +/− 0.005 ns, and 0.300 +/− 0.003 ns. The longer lifetime, τ2, of MDA-MB-231, MDA-MB-157, MCF-12A, and MCF-10A cells were 0.751 +/− 0.013 ns, 0.748 +/− 0.013 ns, 0.894 +/− 0.015 ns, and 0.946 +/− 0.009 ns. The images in Fig. 4 demonstrate that the shorter lifetimes, τ1, are registered from the nucleus region, and the longer lifetimes, τ2, arise from the regions outside the nucleus, including organelles and the cytoplasm. The ratio of the amplitudes, a1/a2, of the two lifetimes, τ1 and τ2, represents the relative contribution of these lifetimes to the overall signal registered from the cells. We also calculated the amplitude-weighted lifetime, τ, which characterizes the mean fluorescence lifetime of the entire cell. The averaged values of τ1, τ2, a1/a2, and τ for each cell line are presented in Fig. 5A–D. They demonstrate that τ1, τ2, and τ are all shorter in the cancer cells as compared to those in the normal cells. As mentioned above, the fluorescence lifetime is inversely related to Fpol^39^. Shorter lifetimes in cancer cells will yield higher Fpol, and longer lifetimes in normal cells will yield lower Fpol. Notably, Fig. 5A–D show that the largest differences between cancer and normal cells were observed for the longer lifetime, τ2. This lifetime characterizes the areas of the cell outside the nucleus. Similarly, the largest differences between Fpol signals, registered from cancer and normal cells, correspond to the organelles and cytoplasm of the cells outside the nucleus. Therefore, the results of our fluorescence lifetime experiments are in complete agreement with the results of fluorescence polarization experiments reported above. Moreover, Fig. 5C, presents greater a1/a2 ratio in normal (MCF-10A and MCF-12A) cells, as compared to cancerous (MDA-MB-231 and MDA-MB-157), indicating greater accumulation of MB in the areas outside the nucleus in cancer cells as compared to normal. This finding is in good agreement with the results of the co-localization experiments, which revealed increased accumulation of MB in the mitochondria and lysosomes of cancer cells.

### Breast cancer cells exhibit increased MB Fpol for a range of dye concentrations

All the experiments reported above were performed with 0.05 mg/ml aqueous MB solutions. To investigate possible impact of the dye concentration on the outcome of the MB Fpol experiments, we have repeated fluorescence polarization and co-localization imaging experiments with the lower 0.01 mg/ml MB concentration. The quantitative images are shown in the supplementary materials in Fig. S1 and summarized in Table S2. The results yielded by both 0.01 mg/ml and 0.05 mg/ml concentrations of the dye are compared in Table 1. They demonstrate that 0.01 and 0.05 mg/ml MB concentrations yield similar results. At both concentrations, larger Fpol difference between cancer and normal cells was detected when nuclei were excluded from the analysis. Moreover, MB subcellular distribution at 0.01 mg/ml concentration parallels our results at 0.05 mg/ml concentration. These findings are summarized in Table 2. In comparison to normal, cancer cells show increased uptake of MB in mitochondria and lysosomes, whereas accumulation of MB in nuclei was comparable among all cell lines. This indicates that the range of MB concentrations between 0.01 and 0.05 mg/ml can be used successfully.

**Table 1:** Fluorescence Polarization of Methylene Blue in Cells. N is the number of the cells.

Cell Line	0.05 mg/ml MB concentration	0.01 mg/ml MB concentration
**MB Fluorescence Polarization of the Cells**		
MDA-MB-231	24.91 × 10^-2^ ± 0.15 × 10^-2^ (N = 103)	25.09 × 10^-2^ ± 0.15 × 10^-2^ (N = 117)
MDA-MB-157	25.16 × 10^-2^ ± 0.14 × 10^-2^ (N = 105)	25.02 × 10^-2^ ± 0.13 × 10^-2^ (N = 100)
MCF-12A	22.29 × 10^-2^ ± 0.14 × 10^-2^ (N = 103)	22.05 × 10^-2^ ± 0.16 × 10^-2^ (N = 113)
MCF-10A	20.88 × 10^-2^ ± 0.12 × 10^-2^ (N = 104)	21.68 × 10^-2^ ± 0.17 × 10^-2^ (N = 111)
**MB Fluorescence Polarization of the Cells Excluding Nuclei**		
MDA-MB-231	23.89 × 10^-2^ ± 0.13 × 10^-2^ (N = 103)	24.77 × 10^-2^ ± 0.12 × 10^-2^ (N = 117)
MDA-MB-157	24.24 × 10^-2^ ± 0.13 × 10^-2^ (N = 105)	24.90 × 10^-2^ ± 0.10 × 10^-2^ (N = 100)
MCF-12A	21.06 × 10^-2^ ± 0.13 × 10^-2^ (N = 103)	21.34 × 10^-2^ ± 0.12 × 10^-2^ (N = 113)
MCF-10A	20.21 × 10^-2^ ± 0.11 × 10^-2^ (N = 104)	21.01 × 10^-2^ ± 0.13 × 10^-2^ (N = 111)
**MB Fluorescence Polarization of the Cell Nuclei**		
MDA-MB-231	25.37 × 10^-2^ ± 0.11 × 10^-2^ (N = 103)	25.54 × 10^-2^ ± 0.10 × 10^-2^ (N = 117)
MDA-MB-157	25.43 × 10^-2^ ± 0.11 × 10^-2^ (N = 105)	25.28 × 10^-2^ ± 0.09 × 10^-2^ (N = 100)
MCF-12A	23.52 × 10^-2^ ± 0.11 × 10^-2^ (N = 103)	24.35 × 10^-2^ ± 0.11 × 10^-2^ (N = 113)
MCF-10A	22.33 × 10^-2^ ± 0.10 × 10^-2^ (N = 104)	23.48 × 10^-2^ ± 0.12 × 10^-2^ (N = 111)

**Table 2:** Co-localization of MB and cell organelles. N is the number of the cells.

Cell Line	0.05 mg/ml MB concentration	0.01 mg/ml MB concentration
**Pearson’s R of MB and Nuclei**		
MDA-MB-231	0.43 ± 0.03 (N = 95)	0.44 ± 0.01 (N = 133)
MDA-MB-157	0.38 ± 0.03 (N = 112)	0.38 ± 0.02 (N = 97)
MCF-12A	0.36 ± 0.03 (N = 65)	0.41 ± 0.01 (N = 114)
MCF-10A	0.35 ± 0.03 (N = 88)	0.34 ± 0.02 (N = 100)
**Pearson’s R of MB and Mitochondria**		
MDA-MB-231	0.35 ± 0.01 (N = 49)	0.39 ± 0.01 (N = 52)
MDA-MB-157	0.34 ± 0.01 (N = 60)	0.40 ± 0.01 (N = 57)
MCF-12A	0.23 ± 0.01 (N = 32)	0.20 ± 0.01 (N = 50)
MCF-10A	0.21 ± 0.01 (N = 42)	0.15 ± 0.01 (N = 46)
**Pearson’s R of MB and Lysosomes**		
MDA-MB-231	0.29 ± 0.02 (N = 50)	0.34 ± 0.01 (N = 75)
MDA-MB-157	0.28 ± 0.02 (N = 59)	0.31 ± 0.01 (N = 67)
MCF-12A	0.22 ± 0.02 (N = 36)	0.20 ± 0.01 (N = 50)
MCF-10A	0.25 ± 0.02 (N = 54)	0.20 ± 0.01 (N = 57)

## Discussion

Great advances have been made in the detection of cancer. However, identification of single cancer cells remains difficult. To achieve accurate cancer diagnosis, researchers have been studying molecular cancer biomarkers, such as oncogenes and oncoproteins^4^. However, out of more than 100 types of cancer, only a few of them at the present time have well-known biomarkers^4^. Moreover, available biomarkers are usually associated with specific tumor subtypes^4^. Therefore, biomarkers could be helpful in selecting proper treatment for different cancer subtypes, but are less suitable for diagnosis. In contrast, our results indicate that MB Fpol imaging may be useful for the detection of several types of cancers. We have shown that increased MB Fpol can be explained by accumulation of positively charged MB in the negatively charged mitochondria in cancer cells. Other studies revealed that the degree of MB accumulation increases with the MMP^27,31^. Since increased MMP has been found in many other cancers, including colon cancer, renal cancer, lung cancer and pancreatic cancer^34^, MB Fpol imaging may provide a useful method for diagnosing these cancers as well. Further studies should explore whether MB Fpol is increased in other types of cancer with high MMP.

Our results demonstrate that a range from at least 0.01 to 0.05 mg/ml MB concentrations yields similar MB Fpol and dye localization. This indicates that our method of breast cancer detection at the cellular level will work for a variety of dye concentration and will not be restricted by specific dye concentration or a demanding staining protocol to achieve that concentration.

A key advantage of MB Fpol imaging is that fluorescence polarization does not depend on the absolute intensity of the fluorescence emission. A reliable interpretation of the fluorescence intensity measurements requires strict control of illumination and staining parameters. Moreover, fluorescence emission is modulated by scattering and absorption properties of the imaged medium. Therefore, imaging techniques based on fluorescence intensity often yield inconsistent results^40–42^.

In this work we observed decreased fluorescence lifetimes of MB in cancer cells as compared to normal cells. This means that MB fluorescence life-time imaging (FLIM) may also be employed for cancer detection. However, FLIM utilizes expensive equipment and sophisticated image processing algorithms. Rapid, robust and simple fluorescence polarization imaging does not depend on a priori assumptions on the properties of the cells and/or extensive image processing. Therefore, it may have an advantage over FLIM in the context of practical applications.

MB Fpol imaging could be used to improve diagnosis of early stage breast cancer. Currently, FNA cytology is based on evaluation of cell morphology. This method is subjective and suffers from low specificity. Increased Fpol from cancer as compared to normal cells may provide specificity at the level of single cells for detecting breast malignancies. An important advantage of the multimodal fluorescence emission and polarization imaging is that the morphology of the cells and quantitative polarization information can be evaluated simultaneously. Moreover, diagnosing carcinoma in breast FNA specimens may be challenging due to the limited amount of the material available^5^. Optical illumination and detection can be conducted via the optical fiber. Therefore, it does not require removal of tissue or cells from the body and, therefore, holds the potential to enable sensitive and high-resolution interrogation of as much tissue material as needed for accurate diagnosis, thus reducing the errors associated with sampling. Our finding that the cells remained viable during and after the staining and imaging procedure points towards the feasibility of an *in vivo* approach to diagnose cancer at the cellular level.

In surgical settings, rapid acquisition of high-contrast and high-resolution optical images of the excisional margins may enable the surgeon to observe cancer cells at the tumor margin in real time. In comparison to other imaging fluorophores MB has been approved by the FDA and has been routinely used in breast cancer surgery for mapping sentinel lymph nodes^26^. The immediate availability of images and high contrast between normal tissue and cancer cells will make it easy for the surgeon to locate the boundaries of the tumor in the operating room without the assistance of a pathologist. Such an approach to image-guided cancer surgery holds the potential to decrease recurrence and re-excision rates.

In summary, we developed a unique quantitative technique for detecting cancer at the cellular level based on MB Fpol imaging of single live cells. We validated our approach by demonstrating significantly higher Fpol of MB in cultured human breast cancer cells relative to normal human breast epithelial cells. We confirmed that our method is accurate, robust, and works for a range of dye concentrations. As our optical technology is simple, safe and nondestructive, it can be readily incorporated into cancer detection and treatment protocols that are currently used, or utilized as a stand-alone technique. In addition, by investigating intracellular localization and fluorescence lifetime of MB, we have obtained evidence that enhanced MB Fpol in cancer cells is due to its increased accumulation in mitochondria and shorter fluorescence lifetime in cancer relative to normal cells.

## Methods

### Cell lines and cell culture

Human breast cancer cell lines, MDA-MB-231 and MDA-MB-157, and two immortalized normal breast epithelial cell lines, MCF-12A and MCF-10A, were obtained from the American Type Culture Collection (ATCC, Manassas, VA). MDA-MB-231 and MDA-MB-157 cells were grown in Leibovitz’s L-15 medium (ATCC, Manassas, VA) supplemented with 10% fetal bovine serum (ATCC, Manassas, VA) and cultured in a humidified CO_2_-free atmosphere at 37 °C. MCF-12A and MCF-10A cells were grown in Dulbecco’s Modified Eagle Medium/Nutrient Mixture F-12 (DMEM/F12, ThermoFisher Scientific, Waltham, MA) supplemented with 20 ng/ml human epidermal growth factor (ThermoFisher Scientific, Waltham, MA), 100 ng/ml cholera toxin (Sigma-Aldrich, St. Louis, MO), 0.01 mg/ml insulin (Sigma-Aldrich, St. Louis, MO), 500 ng/ml 95% hydrocortisone (Fisher Scientific, Hampton, NH), 5% horse serum (Fisher Scientific, Hampton, NH), and cultured in a humidified atmosphere at 37 °C and 5% CO_2_. Each cell line underwent less than ten passages.

### Cell handling and staining

For imaging and analysis, the cells were plated at a density of 25,000 cells/well in 35 mm glass bottomed cell culture dishes (*In vitro* scientific, Mountain View, CA) and cultured overnight. For MB Fpol and fluorescence lifetime imaging (FLIM) experiments, cells were transported to the Advanced Biophotonics Laboratory and Tufts University, respectively. During transportation, cells were kept in Leibovitz’s L-15 medium (ATCC, Manassas, VA) at 37 °C. Prior to staining and imaging, cells were allowed to rest for 40 min at 37 °C and relative humidity (RH) of 95% for recovery. Then the cell layers were incubated with 0.05 mg/ml aqueous solution of MB (Akorn, Inc., Lake Forest, IL) (0.5 ml per well) for 20 min. After staining, cells were rinsed three times with phosphate buffered saline (PBS, Fisher Scientific, Hampton, NH).

### Confocal fluorescence polarization (Fpol) imaging and analysis

**Figure 6:**
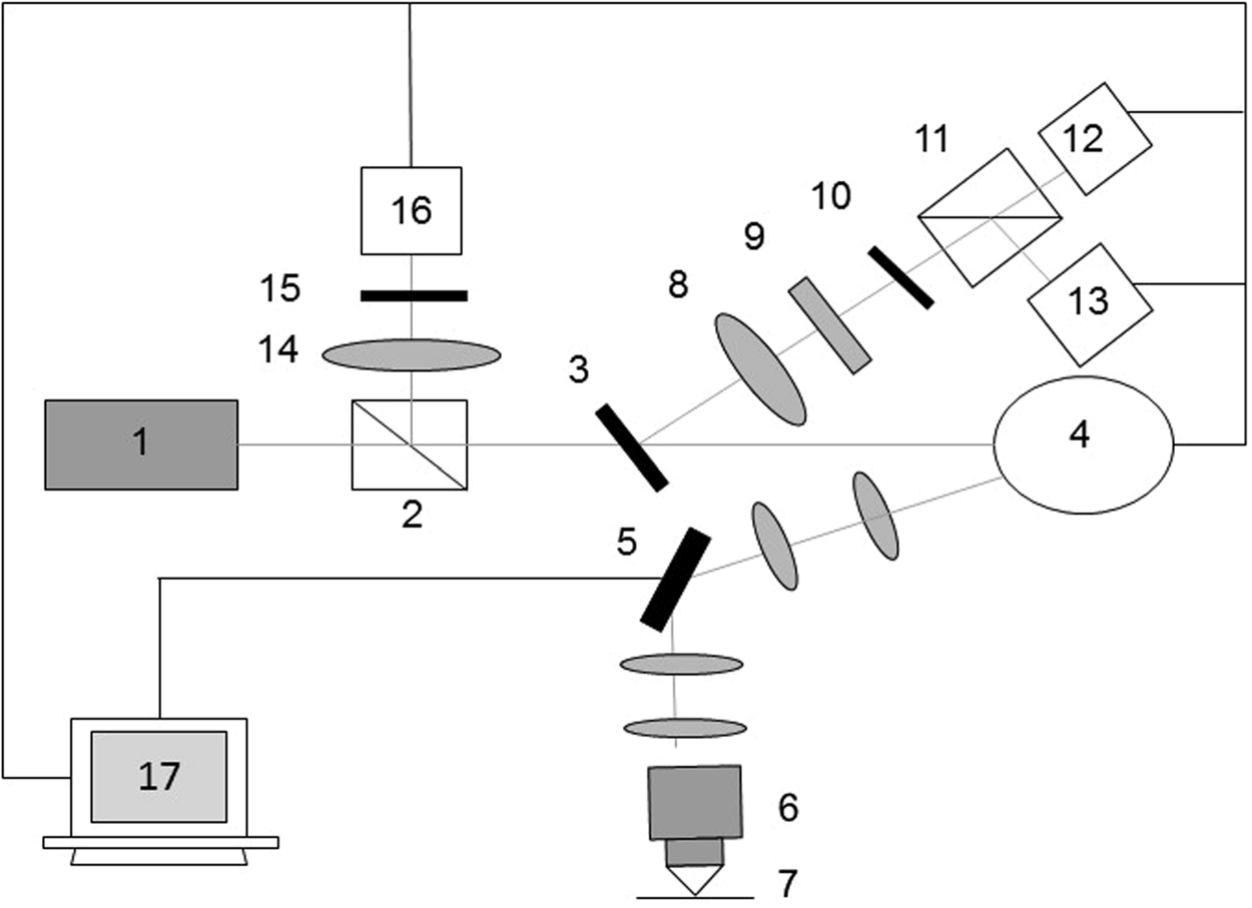
Schematic of the point scanning confocal fluorescence polarization imaging system. 1 – laser, 2 – beam splitter, 3 – dichroic mirror, 4 – polygon mirror, 5 – galvanometric mirror, 6 – objective, 7 – sample plane, 8 – focusing lens, 9 – fluorescence filter, 10 – pinhole, 11 – polarizing beam splitter, 12 – PMT for cross-polarized fluorescence, 13 – PMT for co-polarized fluorescence, 14 – focusing lens, 15 – pinhole, 16 – PMT for reflectance, 17 – computer.

Imaging was performed using an in-house built multimodal confocal imaging system, presented in Fig. 6. Vertically polarized light from a 642 nm diode laser (Micro Laser Systems, Garden Grove, CA) was used for illumination. The laser beam was scanned in x and y directions using a polygon mirror (Lincoln Laser, Phoenix, AZ) and a galvanometric mirror (General Scanning Inc., Billerica, MA), respectively. The scanning rate was 7 frames per second. The laser beam was focused onto the imaging plane by a 63X/1.4NA oil immersion objective (Carl Zeiss, Oberkochen, Germany). Fluorescence light emitted from the sample, was reflected by a 12-degree dichroic mirror (Iridian Spectral Technologies, Ottawa, Ontario) and focused onto a 100 µm pinhole. A 690 nm bandpass filter with a full width at half maximum of 20 nm (Chroma Technology Corp., Bellows Falls, VT) was placed before the pinhole to further reject the excitation light. Fluorescence emission co- and cross-polarized with respect to the incident laser light was separated by a polarizing beam splitter (Karl Lambrecht Co., Chicago, IL), and simultaneously registered by the two photomultiplier tubes (PMTs) (Hamamatsu Photonics, Shizuoka Pref., Japan). The elastically scattered light passed through a dichroic mirror, was deflected by a non-polarizing beam splitter (Tower Optical, Boynton Beach, FL), and focused onto a 200 µm pinhole of the reflectance PMT (Hamamatsu Photonics, Shizuoka Pref., Japan). Signals were recorded as 8-bit gray-scale images. The system yielded lateral resolution better than 0.9 µm and axial resolution of 3 µm. We characterized the sensitivity of the fluorescence detection channels of our imager to the vertical (co-polarized) and horizontal (cross-polarized) polarization of light by measuring the G-factor following the methodology of Seigel *et al*.^43^. We determined the G-factor of our imaging system to be 0.75.

Fpol analysis was performed in MetaMorph imaging software (Molecular Devices, Sunnyvale, CA). First, images were thresholded (low: 2; high: 254) to remove the background and saturated pixels. Average intensity values of each cell in co- and cross-polarized fluorescence images (*I*_*co*_ and *I*_*cross*_) were generated, and *Fpol* was calculated using eq. 1:

1 $$Fpol=\frac{{I}_{co}-G\times {I}_{cross}}{{I}_{co}+G\times {I}_{cross}},$$

where *G* is G-factor of the system.

For quantitative fluorescence polarization imaging the multimodal confocal system was calibrated using MB solutions with different viscosities. In particular, we used 0.05 mg/ml homogeneous MB solutions in PBS (Fisher Scientific, Hampton, NH) and in glycerol (Fisher Scientific, Hampton, NH). To check the fidelity of quantitative Fpol imaging using our confocal imaging system, the values of fluorescence polarization of the same mixtures were determined using a commercial polarization-sensitive fluorometer (FluoroMax-4, Horiba, Edison, NJ). For both solutions, the Fpol value obtained using confocal imaging system agreed well with those measured using fluorometer. Fluorescence polarization values obtained for water/glycerol MB mixes were used in the course of the project as a reference for calibrating the system and confirming the fidelity of fluorescence polarization imaging before and after the cell experiments.

Fpol images were generated in ImageJ available at https://imagej.nih.gov/ij/. The procedure was adapted from a previously published protocol^43^. Fluorescence emission (I_co_ + G × I_cross_) and difference (I_co_ − G × I_cross_) images were generated using Image Calculator plugin. Fpol image was generated using Ratio Plus plugin. NucMed plugin was used to assign pseudo colors to the images. Color range was set from 0 to 0.34.

### MB subcellular localization

Cell monolayers were first incubated for 30 min with 100 nM LysoTracker Yellow HCK-123 (Life Technologies, Woburn, MA) for staining of lysosomes or 50 nM MitoTracker Green FM (Life Technologies, Woburn, MA) for staining of mitochondria. Then the cells were rinsed with PBS and incubated with MB under the same conditions as in the Fpol experiments, followed by 5 min incubation with a 2 µg/ml Hoechst-33342 (Life Technologies, Woburn, MA), which stains the nuclei. Imaging was performed on a confocal microscope (FV1000, Olympus, Shinjuku, Tokyo, Japan) with a 60X/1.2 NA PLANAPO objective lens (Olympus, Shinjuku, Tokyo, Japan). The system allowed for simultaneous acquisition of four channels. A 405 nm laser diode (for Hoechst-33342), a 488 nm line of an argon ion laser (for LysoTracker Yellow/ MitoTracker Green), and a 635 laser diode (for MB) were used for excitation. Fluorescence signal passed through a 405/488/559/635 dichroic mirror, and separated into three channels. Fluorescence of Hoechst-33342 was reflected by a 490 nm dichroic mirror, passed through a 425 ± 25 nm bandpass filter, and collected by the first PMT. Fluorescence of LysoTracker Yellow or MitoTracker Green was reflected by a 560 nm dichroic mirror, passed through a 500 ± 50 nm bandpass filter, and collected by the second PMT. MB fluorescence signal was reflected by a 650 nm dichroic mirror, passed through a 705 ± 50 nm bandpass filter, and collected by the third PMT. A transmission differential interference contrast image was acquired by the fourth PMT. It was displayed in grayscale. Figs S2–S4 in the supplementary materials show example images of co-localization experiments where cells were stained with MB, mito-tracker, lyso-tracker, and DAPI. For presentation, Hoechst-33342 images were displayed in blue, LysoTracker and MitoTracker images were displayed in green, and MB images were displayed in red. Quantification of co-localization between MB and each organelle was performed using the Coloc 2 plugin in ImageJ. ROIs were selected to outline the investigated organelles. Pearson’s R coefficients were determined using the Coloc2 analysis.

### Fluorescence lifetime imaging (FLIM) experiments and data analysis

Two-photon excitation fluorescence (TPEF) and FLIM was performed using a Leica TCS SP8 confocal microscope (Leica Microsystems, Wetzlar, Germany). Excitation was performed at 900 nm with a mode-locked Ti:Sapphire laser (Insight DS + , Spectra-Physics, Santa Clara, CA), focused with a water immersion 40X/1.10 NA Leica HC PL APO objective lens (Leica Microsystems, Wetzlar, Germany). TPEF images were collected over a 0.29 × 0.29 mm field of view with 512 × 512 binning, and imaged through a pinhole aperture and a dispersive spectral detection system onto a hybrid avalanche photodiode detector (Leica HyDTM, Wetzlar, Germany). The spectral bandwidth of the HyD detector was set at 685–710 nm and the pinhole was opened to 7.8 AU. For FLIM measurements, the signal from the HyD detector was fed into a time-correlated single photon counting module (PicoHarp 300, PicoQuant Inc., Berlin, Germany) capable of measuring fluorescence lifetimes as short as 70 ps.

SymphoTime64 software (PicoQuant Inc., Berlin, Germany) was used to analyze the FLIM data. Fluorescence lifetimes were determined for each cell. Instrument response function (IRF) was reconstructed in the software. Fluorescence lifetimes (τ1, τ2) and the corresponding amplitudes (a1, a2) were generated by fitting the fluorescence decay with a bi-exponential reconvolution model. The quality of the fit was considered acceptable when χ^2^ was between 1 and 1.5. Amplitude-weighted lifetime, τ, was calculated using eq. 2:

2 $$\tau =\frac{a1\times \tau 1+a2\times \tau 2}{a1+a2}.$$

### Cell viability test

To determine cell viability after imaging experiments we used trypan blue^44^. Specifically, we added 0.5 ml of 0.4% trypan blue (Sigma-Aldrich, St. Louis, MO) per well. The cell mono-layers were stained for 1 min. After staining, the cells were rinsed three times with PBS imaged and counted under the light microscope (PrimoVert, Carl Zeiss Microscopy, Peabody, MA) equipped with using 20X/0.3 NA PlANAPO objective lens (Zeiss, Oberkochen, Germany). Supplementary Fig. S5 presents example bright field images of dead and live cells after trypan blue staining. MB stained cells were light blue. The cells stained with both MB and trypan blue were purple and were considered dead.

### Statistical analysis

For statistical evaluation of the results, estimates of the means and standard deviations of Fpol values were obtained in the fluorescence polarization imaging experiments, Pearson’s R coefficients were obtained in MB localization experiments, and the lifetimes obtained in fluorescence life-time experiments were obtained for each cell line. The data were statistically evaluated using a mixed effects linear model^45^. The model predicted the outcomes measured as a function of the fixed effect of cell line and random effects to account for the inherit correlation in the repeated observations within the same sampling unit. Least Square Estimates of the means and standard errors were obtained for each cell line. The significance of the differences between the cancer and normal cell lines was assessed. *P* < 0.001 was considered significant.

## Supplementary information

Supplementary Information
