# Supplementary material for: Fluorescence Polarization of Methylene Blue as a Quantitative Marker of Breast Cancer at the Cellular Level
Source: Sci Rep. 2019 Jan 30;9:940. doi: 10.1038/s41598-018-38265-0 (PMC6353996; doi:10.1038/s41598-018-38265-0)
Supplement: Supplementary file 1 — Supplementary Information [file 41598_2018_38265_MOESM1_ESM.pdf]

Fluorescence Polarization of Methylene Blue as a Quantitative Marker of Breast Cancer at the Cellular Level

Anna N Yaroslavsky,[1, 2]* Xin Feng,[1] Alona Muzikansky,[3] Michael R Hamblin[2]

Anna_Yaroslavsky@uml.edu

Phone: 978-934-1350

Fax: 978-934-1305

[1] Advanced Biophotonics Laboratory, Department of Physics and Applied Physics, University of Massachusetts, Lowell, 1 University Ave., Lowell, MA 01854

[2] Wellman Center for Photomedicine, Massachusetts General Hospital**,** Harvard Medical School, 40 Blossom Street, Boston, MA 02114

[3] Massachusetts General Hospital Biostatistics Center, Massachusetts General Hospital**,** 50 Staniford Street, Boston, MA 02114

SUPPLEMENTARY INFORMATION

Quantitative MB Fpol of All the Investigated Cells

In total, we have imaged MB fluorescence emission and polarization from 2833 cells, including 1482 cancer cells (563 cells from MDA-MB-231 cell line and 919 cells from MDA-MB-157 cell line) and 1351 normal cells (700 cells from MCF-10A cell line and 651cells from MCF-12A cell line). However, only 856 cells, including 425 cancer cells (220 cells from MDA-MB-231 cell line and 205 cells from MDA-MB-157 cell line) and 431 normal cells (215 cells from MCF-10A cell line and 216 cells from MCF-12A cell line), with clearly identified nuclei were selected for segmentation and subcellular fluorescence polarization analysis. The results obtained for these 856 cells are presented in the manuscript. Supplementary Tables 1 and 2 summarize quantitative MB Fpol obtained for all the 2833 imaged cells at 0.05 mg/ml and 0.01 mg/ml concentrations, respectively. The Fpol results obtained for all the imaged cells compare well with those presented in the manuscript.

<u>Supplementary Table S1</u>. Comparison of MB Fluorescence Polarization (Fpol) Exhibited by Breast Cancer and Normal Cells at 0.05 mg/ml Concentration of the Dye

Cell Line	*# of Cells*	*Whole Cell Fpol*		*Difference in Fpol*		*P Value*	
		Averaged Fpol	*Standard Deviation*	*versus MCF-10A*	*versus MCF-12A*	*versus MCF-10A*	*versus MCF-12A*
MDA-MB-231	245	25.15×10^{-2}	0.09×10^{-2}	22%	14%	<0.0001	<0.0001
MDA-MB-157	402	24.95×10^{-2}	0.08×10^{-2}	21%	13%		
MCF-10A	310	20.58×10^{-2}	0.08×10^{-2}				
MCF-12A	253	22.06×10^{-2}	0.09×10^{-2}				

<u>Supplementary Table S2</u>. Comparison of MB Fluorescence Polarization (Fpol) Exhibited by Breast Cancer and Normal Cells at 0.01 mg/ml Concentration of the Dye

Cell Line	*# of Cells*	*Whole Cell Fpol*		*Difference in Fpol*		*P Value*	
		Averaged Fpol	*Standard Deviation*	*versus MCF-10A*	*versus MCF-12A*	*versus MCF-10A*	*versus MCF-12A*
MDA-MB-231	318	25.20×10^{-2}	0.08×10^{-2}	19%	15%	<0.0001	<0.0001
MDA-MB-157	517	25.09×10^{-2}	0.06×10^{-2}	18%	15%		
MCF-10A	390	21.26×10^{-2}	0.08×10^{-2}				
MCF-12A	398	21.90×10^{-2}	0.08×10^{-2}				

Quantitative MB Fpol Images of Cells Incubated with 0.01 mg/ml MB

To demonstrate that the observed MB Fpol differences between cancer and normal cells are similar for a range of the dye concentrations between 0.01 mg/ml and 0.05 mg/ml, we present a Supplementary Fig. 1. It shows fluorescence emission (Figs. 1 A, B, C, D) and Fpol (Figs. 1 E, F, G, H) distribution within the cell lines investigated at 0.01 mg/ml concentration. Comparison to the Fig. 1 of the manuscript confirms that dye uptake and the values of Fpol are similar for both concentrations.

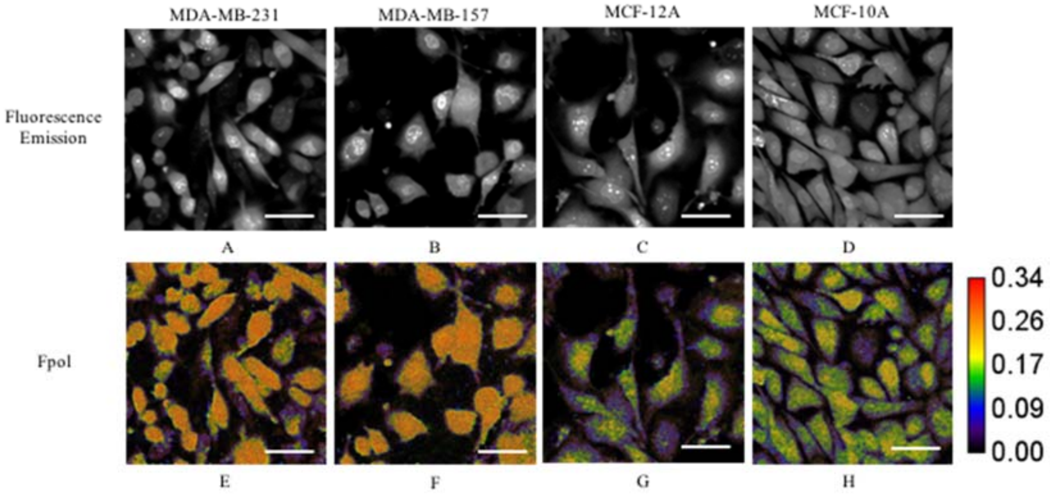

Supplementary Fig. S1 Example MB fluorescence emission and quantitative pseudocolored Fpol images of MDA-MB-231, MDA-MB-157, MCF-12A, MCF-10A cells at 0.01 mg/ml. A-D: fluorescence emission images; E-H: pseudocolored Fpol images. Bar: 50 µm.

Example Images of Co-localization Experiments

Supplementary Figs. S2-S4 show example images of co-localization experiments where cells were stained with MB, mito-tracker, lyso-tracker, and DAPI. The degree of co-localization between MB and each tracker was determined from these images using Pearson’s R coefficients.

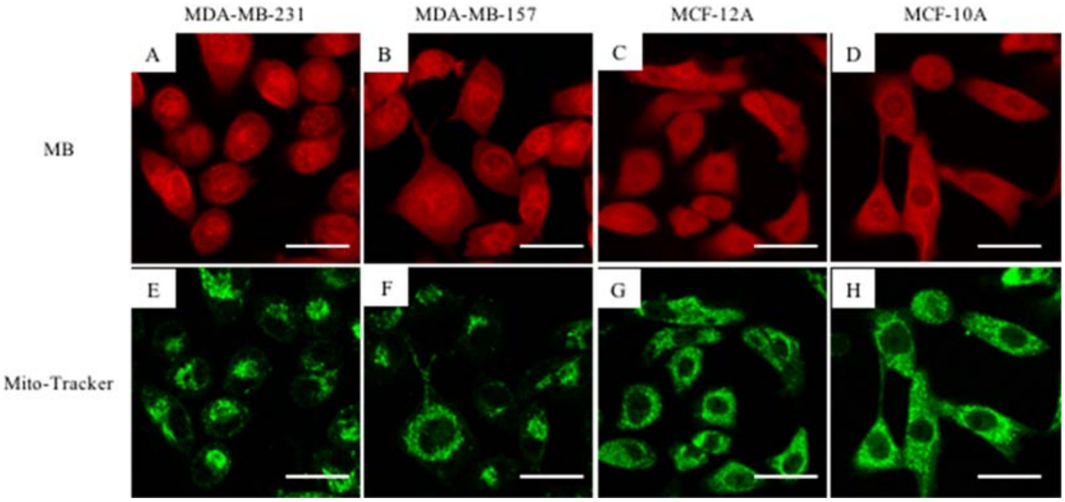

Supplementary Fig. S2 Example images of a representative co-localization experiment. (A-D) MB fluorescence emission images; (E-H) fluorescence emission images of mito-tracker. Bar = 30 µm.

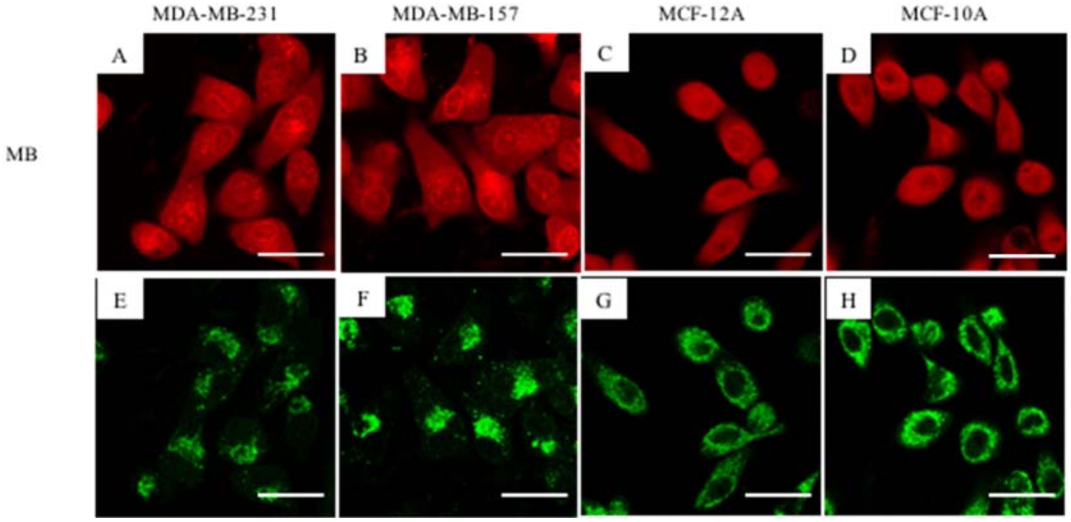

Supplementary Fig. S3 Example images of a representative co-localization experiment. (A-D) MB fluorescence emission images; (E-H) fluorescence emission images of lyso-tracker. Bar = 30 µm.

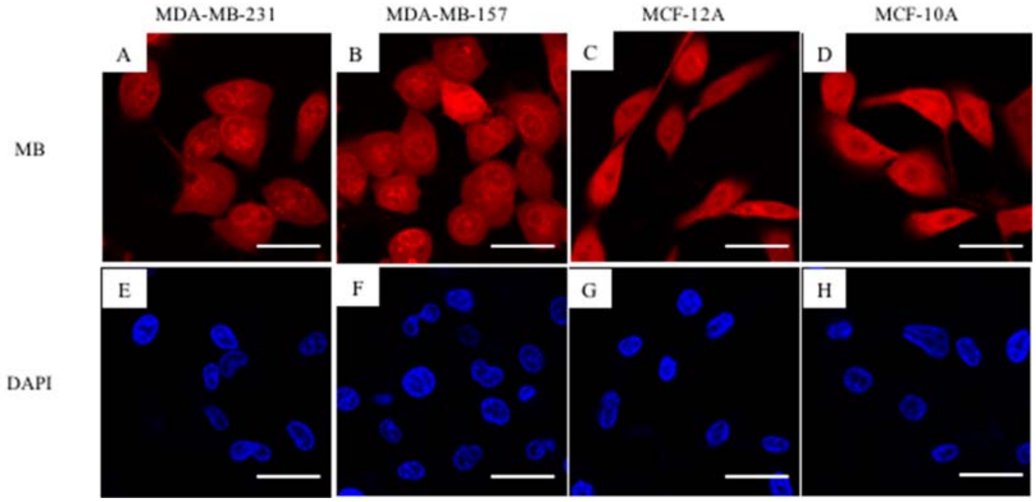

Supplementary Fig. S4 Example images of a representative co-localization experiment. (A-D) MB fluorescence emission images; (E-H) fluorescence emission images of DAPI. Bar = 30 μm.

Viability Test

To show that the MB stained cells that uptake trypan blue (dead cells) can be easily visually distinguished from the MB stained cells that don't uptake trypan blue (live cells) we present supplementary Fig. S5 with example bright field images of dead and live cells after trypan blue staining, respectively. Dead cells are purple because they uptake both MB and trypan blue. Live cells are light blue because they uptake only MB.

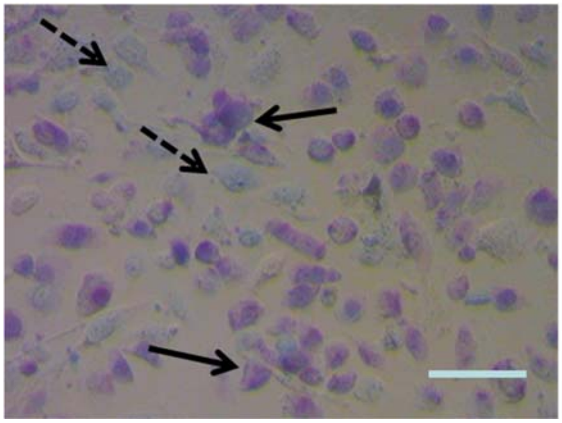

Supplementary Fig. S5 Bright field images of dead (solid arrows) and live (dotted arrows) cells after methylene blue and trypan blue staining. Bar = 50 μm.
